# Noise pollution in the ICU: time to look into the mirror

**DOI:** 10.1186/s13054-014-0493-1

**Published:** 2014-08-27

**Authors:** Koen S Simons, Munhum Park, Armin Kohlrausch, Mark van den Boogaard, Peter Pickkers, Werner de Bruijn, Cornelis PC de Jager

**Affiliations:** Department of Intensive Care and Emergency Medicine, Jeroen Bosch Ziekenhuis, Henri Dunantstraat 1, ‘s Hertogenbosch, 500 ME The Netherlands; Department of Intensive Care Medicine, Radboud University Medical Centre, Geert Grooteplein 21, Nijmegen, 6500 HB The Netherlands; Smart Sensing & Analysis Group, Philips Research Laboratories, High Tech Campus 36, Eindhoven, 5656 AE The Netherlands

We read with interest the recent issue of *Critical Care* in which Darbyshire and Young [1] reported on noise levels in five different ICUs and demonstrated average sound pressure levels far above the World Health Organization recommended standard of 35 dB L_Aeq_ (A-weighted energy-equivalent sound pressure level in decibels). Although their article provides an interesting insight into the ICU soundscape, the authors did not attempt to investigate the sources of noise. In the literature, only few studies have performed an analysis of noise sources, using either questionnaires [2] or a human observer in the patient’s room [3–5]. Aiming to provide more insight into this matter, some of the authors recently performed an acoustic survey in an ICU room in order to determine which sources are responsible for the high noise levels, and details of this study were recently published [6]. Briefly, an audio recording was made by using a calibrated microphone in an ICU room at Jeroen Bosch Hospital for a duration of 67 hours. In addition to the analysis of various acoustic parameters, a 24-hour audio fragment was manually annotated by six research assistants. All sound events (n = 27,421) were identified by using 28 noise source labels, which were grouped into five noise categories.

Acoustic analysis showed an average sound pressure level of 61 dB L_Aeq_ when the room was occupied. In agreement with the aforementioned study, the number of predicted loudness peaks was up to 90 per hour. Restorative periods were defined as periods of at least 5 minutes in which the sound pressure level relative to the background level did not exceed 17.7 dBA (A-weighted sound pressure level in decibels); only approximately 46% of the periods recorded at night were considered to be restorative, and the average duration of these restorative periods was approximately 13 minutes. Source-specific analysis revealed that, on average, noisy events related to staff activities (54 dB L_Aeq_) occurred approximately 10 times per minute, staff speech (55 dB L_Aeq_) occurred approximately 4 times per minute, and alarms (57 dB L_Aeq_) also occurred approximately 4 times per minute. Further analyses showed that 57% of total acoustic energy and 92% of predicted loudness peaks could be attributed to the activities and speech of hospital personnel (Figure 1). We agree with Darbyshire and Young [1] that high sound pressure levels may have detrimental effects in the already vulnerable population of ICU patients. The aforementioned study demonstrates that more than half of all acoustic energy in an ICU is related to human activities and speech and therefore is potentially modifiable. Strategies involving the adaptation of human behavior therefore may prove to be very effective at reducing noise pollution in the ICU.

**Figure 1:**
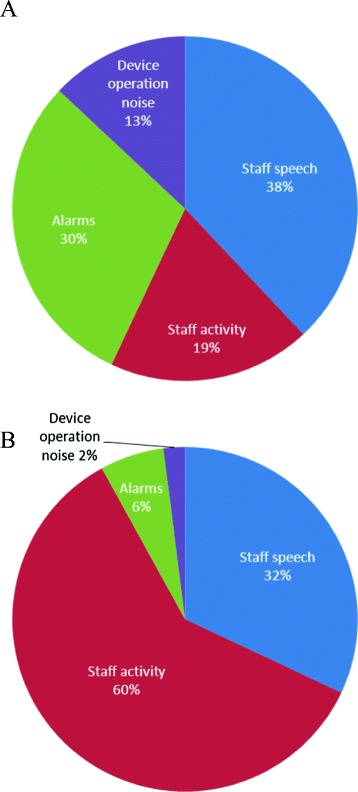
**The contribution of each noise category for (A) the acoustic energy and (B) the number of predicted loudness peaks.** Noise generated by or involving patients is excluded. For more details, refer to [6].

## Abbreviation

dB L_Aeq_: A-weighted energy-equivalent sound pressure level in decibels
